# Impact of psychiatric disorders on the hemodynamic and quality of life outcome of balloon pulmonary angioplasty in patients with chronic thromboembolic pulmonary hypertension: a retrospective study

**DOI:** 10.1186/s12931-023-02579-z

**Published:** 2023-11-11

**Authors:** Kazutoshi Hirose, Shun Minatsuki, Akihito Saito, Hiroki Yagi, Norifumi Takeda, Masaru Hatano, Issei Komuro

**Affiliations:** 1 Department of Cardiovascular Medicine, Graduate School of Medicine,, University of Tokyo, 7-3-1, Hongo, Bunkyo-ku Tokyo, 113-8655 Japan; 2 International University of Health and Welfare, Tokyo, Japan

**Keywords:** Chronic thromboembolic pulmonary hypertension, Balloon pulmonary angioplasty, Psychiatric disorder, Quality of life

## Abstract

**Background:**

Balloon pulmonary angioplasty (BPA) has beneficial effects on pulmonary hemodynamics, exercise capacity, and quality of life (QOL) in patients with chronic thromboembolic pulmonary hypertension (CTEPH). Recently, emerging evidence suggests a relationship between CTEPH and psychiatric disorders (PD). However, data on the clinical efficacy of BPA in CTEPH patients with PD are lacking.

**Methods:**

We retrospectively analyzed 75 patients with inoperable/residual CTEPH who underwent BPA and right-sided heart catheterization before the initial BPA and within 1 year after the last procedure. QOL was evaluated using the European Quality of Life Five Dimension (EQ-5D) scale in 27 patients before and after BPA sessions. Baseline and post-procedural hemodynamic, functional, and QOL parameters were compared between the patients with and without PD.

**Results:**

Among the 75 participants, 22 (29.3%) patients were categorized in the PD group. Although PD group had a similar mean pulmonary artery pressure level compared with non-PD group (40 ± 7 vs. 41 ± 9 mmHg, p = 0.477), they tended to have unfavorable QOL status (0.63 ± 0.22 vs. 0.77 ± 0.19, p = 0.102). BPA significantly improved pulmonary hemodynamics, laboratory parameters and exercise tolerance in both groups. BPA also significantly improved EQ-5D scores in the non-PD group (from 0.77 ± 0.19 to 0.88 ± 0.13, p < 0.001), but the scores remained unchanged in the PD group (from 0.63 ± 0.22 to 0.67 ± 0.22, p = 0.770). During the long-term period [1,848 (1,055–2,565) days], both groups experienced similar mortality rates (PD 4.6% vs. non-PD 5.7%, p = 1.000).

**Conclusions:**

BPA improved hemodynamic and functional parameters irrespective of PD, but its effect on QOL was limited in patients with PD.

## Background

Balloon pulmonary angioplasty (BPA) has beneficial effects on pulmonary hemodynamics, exercise capacity, and further quality of life (QOL) in patients with inoperable/residual chronic thromboembolic pulmonary hypertension (CTEPH) [1–4]. Chronic unresolved pulmonary embolism is a key manifestation of CTEPH [5, 6], and psychiatric disorders (PD) is a significant risk factor for venous thromboembolism (VTE) [7, 8]. In fact, patients with CTEPH exhibited a higher prevalence of concomitant PD than patients with pulmonary arterial hypertension or the general population [9], indicating a relationship between CTEPH and PD. Recent studies also demonstrated that patients with co-existing PD had reduced QOL and higher mortality rate after pulmonary endarterectomy (PEA) compared with those without PD [10, 11]. Despite the favorable impact of BPA on hemodynamic and QOL status [12, 13], data on the clinical efficacy of BPA in CTEPH patients with PD are lacking. The present study therefore aimed to investigate the hemodynamic, functional, and prognostic effects of BPA in patients with CTEPH, focusing on concomitant PD.

## Methods

### Study population

We retrospectively analyzed 80 consecutive patients with CTEPH undergoing BPA who were inoperable or had residual pulmonary hypertension (PH) after PEA at the University of Tokyo Hospital between April 2008 and March 2023. Five patients were excluded because of a lack of hemodynamic data before or after the BPA procedure. Patients were diagnosed with CTEPH based on co-existing PH assessed by right-sided heart catheterization (RHC), defined as mean pulmonary artery pressure (mPAP) > 20 mmHg with a normal mean pulmonary capillary wedge pressure ≤ 15 mmHg and pulmonary vascular resistance (PVR) > 2 Wood Units [14], and the demonstration of organic pulmonary thromboembolism using contrast lung computed tomography, pulmonary perfusion scintigraphy, and pulmonary angiography. Patients were considered as psychotic according to a previous diagnosis by a psychiatrist, but patients treated for insomnia alone were not defined as having a PD. The end date of the study was March 31, 2023. The investigation conformed to the principles outlined in the Declaration of Helsinki. The need for written informed consent was waived because of the retrospective nature of the study design and minimal risk to patients. This study was approved by the Institutional Review Boards of the University of Tokyo (No 2650).

### Examinations

RHC was performed before the initial BPA (pre-BPA) and within one year after the last BPA (post-BPA). Right atrial pressure, PAP, and pulmonary capillary wedge pressure were measured using a Swan-Gantz catheter (Edwards Life Science, Irvine, CA, USA). Cardiac output was determined with the thermodilution method, and PVR was then calculated. World Health Organization functional score, brain natriuretic peptide (BNP) levels, respiratory function, and 6-min walk test were also assessed at the time of hospitalization for RHC.

### BPA procedure

The BPA procedure has been described previously [15]. Briefly, we introduced a 6-Fr long sheath into the main pulmonary artery with a 0.035-inch guidewire through an 8-Fr sheath inserted in the femoral or internal jugular vein, and selected an individual segmental pulmonary artery using a 6-Fr guiding catheter. The target lesion was visualized by injecting contrast medium during inspiration, and a 0.014-inch guidewire was crossed through the lesion and an optimal-sized balloon (2.0–8.0 mm), determined by angiographic or intravascular ultrasound findings, was inflated. We treated ring-like, web, subtotal, and total lesions. We used a smaller balloon at the first session for each lung, to avoid reperfusion pulmonary edema, followed by a larger balloon from the second session, because of dilated vessels with increased blood flow through the first session. The initial goal of BPA was to achieve an mPAP < 30 mmHg on the basis of the previous studies [2, 16], and additional BPA was performed in patients with residual PH symptoms.

### QOL evaluation

We evaluated QOL using the European Quality of Life Five Dimension (EQ-5D) scale [17], as an established tool for the quantitative assessment of QOL in various clinical settings including psychiatric disorders [18, 19]. The scale consists of five questionnaires (mobility, self-care, usual activities, pain discomfort, and anxiety depression) with five responses (no problems, slight problems, moderate problems, serious problems, and extreme problems) represented by scores of 1–5, respectively, for the individual questions (i.e., no problems = 1, slight problems = 2, moderate problems = 3, serious problems = 4, and extreme problems = 5). The final EQ-5D score was then calculated from the combined scores. The EQ-5D score in Japan ranges from − 0.025 to 1.000, with a higher score indicating a more-favorable health status [17]. We assessed the EQ-5D scores at the time of RHC before and after a series of BPA sessions.

### Statistical analysis

Continuous variables were expressed as mean ± standard deviation or median (interquartile range) and compared between the PD and non-PD groups using unpaired Student’s *t*-tests or Wilcoxon’s rank sum tests, depending on the distribution of the data. Categorical variables were described as number (percentage) and analyzed by χ^2^ or Fisher’s exact tests. Paired *t*-tests or Wilcoxon’s signed-rank tests were used to compare hemodynamic, laboratory, functional, and QOL-related parameters as appropriate. All analyses were carried out using JMP Pro 17 statistical software (SAS Institute, Inc., Cary, NC, USA) and p < 0.05 was considered statistically significant.

## Results

**Fig. 1:**
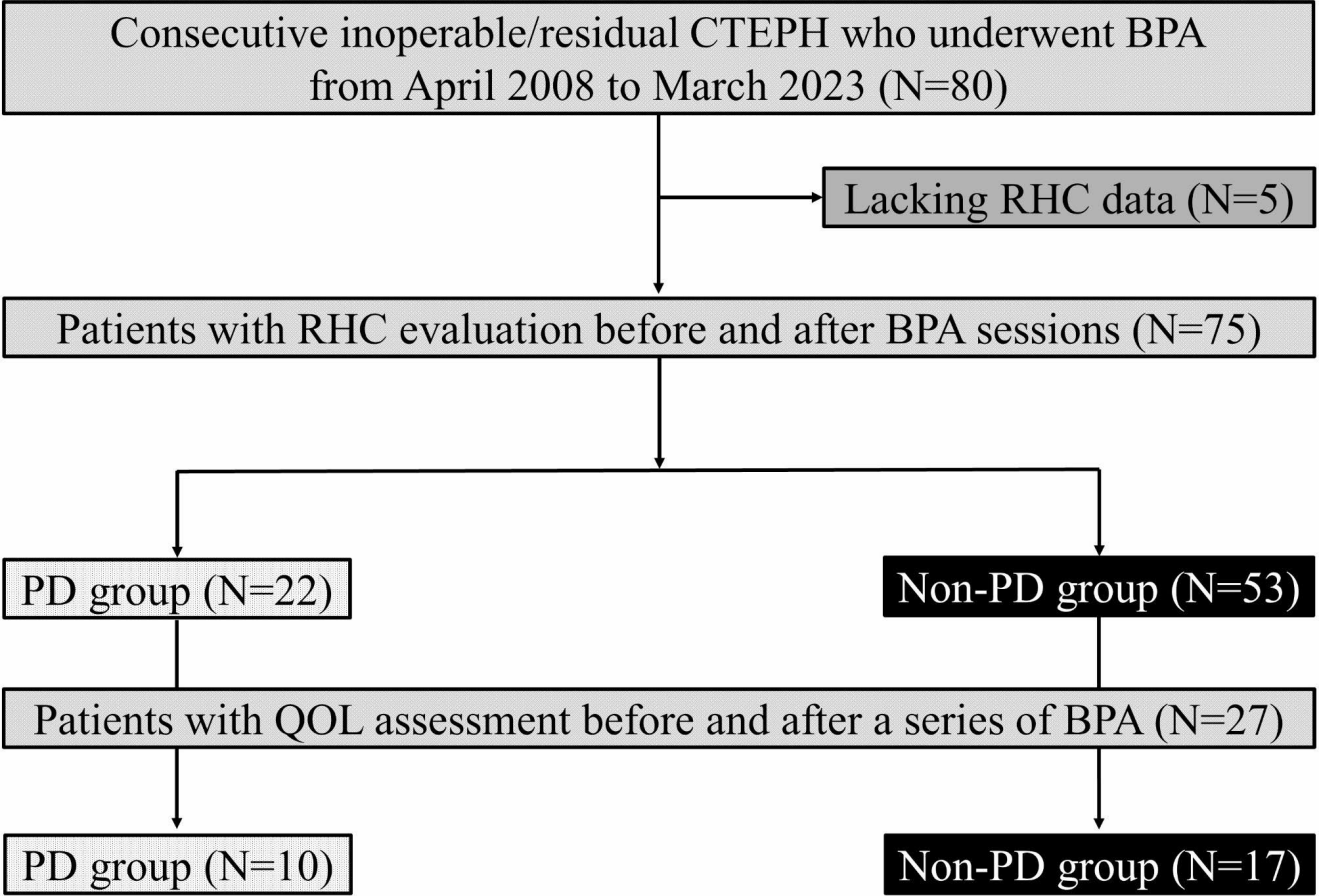
Flow chart of the study. BPA, balloon pulmonary angioplasty; CTEPH, chronic thromboembolic pulmonary hypertension; PD, psychiatric disorder; QOL, quality of life; RHC, right-sided heart catheterization

Seventy-five patients with CTEPH who underwent BPA and follow-up RHC were categorized into the PD group (n = 22) or non-PD group (n = 53) (Fig. 1). The mean age of the overall study population was 62 ± 14 years and 50 (66.7%) patients were women. Thirty-seven (49.3%) patients had a previous history of acute VTE, and 41 (54.7%) patients were diagnosed with CTEPH within 1 year from the onset of symptoms. During the median follow-up period of 1,848 (1,055–2,565) days, the median number of BPA sessions was six (4–8) and four (5.3%) patients died (2 right-sided heart failure, 1 lung cancer, and 1 respiratory infection), with no significant difference in mortality between the PD and non-PD groups (4.6% vs. 5.7%, p = 1.000).

### Prevalence and characteristics of PD in patients with CTEPH treated with BPA

Twenty-two of the 75 patients (29.3%) had PD (Fig. 1). The mean age at diagnosis of PD was 35 ± 13 years. The PD group included seven cases (31.8%) of schizophrenia, six (27.3%) of depression, six (27.3%) of bipolar disorder, ten (45.5%) others, and seven patients (31.8%) with overlap disease concomitant with multiple mental disorders. Most PD patients received antipsychotic agents, while nine patients in the non-PD group received prescribed sleeping pills [21 (95.5%) vs. 9 (17.0%), p < 0.001]. Most patients with PD received a combination of antipsychotic agents [median number of antipsychotic agents: 5 (3–6)] (Table 1). Patients with PD tended to be younger (58 ± 12 vs. 64 ± 15 years, p = 0.060) and had a higher body mass index (25.7 ± 4.4 vs. 22.3 ± 4.5 kg/m^2^, p = 0.002) than those without PD, while both groups had similar smoking status (p > 0.10) (Table 1). The prevalence of acute VTE was comparable between PD and non-PD groups (54.6% vs. 47.2%, p = 0.561), showing similar adherence to anticoagulants in both groups (Table 1). The baseline functional parameters, laboratory examinations, hemodynamic data, and respiratory functions are presented in Table 2. Functional parameters and exercise capacity were comparable between the two groups, but BNP was lower in the PD group [32 (13–105) vs. 117 (54–373) pg/ml, p = 0.001]. Regarding hemodynamic measurements, RHC showed similar mPAP between the two groups (40 ± 7 vs. 41 ± 9 mmHg, p = 0.477), whereas patients with PD tended to have a lower PVR [491 (376–654) vs. 667 (435–792) dyne·s·cm^− 5^, p = 0.066] and mean blood pressure (82 ± 13 vs. 89 ± 16 mmHg, p = 0.086) (Table 2). As for respiratory function, arterial oxygen saturation was significantly lower in the PD group (SaO_2_, 87.9 ± 4.9 vs. 90.9 ± 4.1%, p = 0.009), and patients in the PD group were likely to have lower percent diffusing capacity for carbon monoxide (%DLCO, 75.1 ± 13.1 vs. 82.5 ± 16.4%, p = 0.074) and higher alveolar-arterial oxygen gradient (A-aDO_2_, 46 ± 9 vs. 42 ± 10 mmHg, p = 0.103) (Table 2).

**Table 1:** Baseline characteristics of patients with and without PD

	PD (n = 22)	Non-PD (n = 53)	p value
Age, years	58 ± 12	64 ± 15	0.060
Female, n (%)	16 (72.7)	34 (64.2)	0.473
Body mass index, kg/m^2^	25.7 ± 4.4	22.3 ± 4.5	0.002
Current/past smoker, n (%)	12 (54.6)	22 (41.5)	0.302
History of acute VTE, n (%)	12 (54.6)	25 (47.2)	0.561
Poor adherence to anticoagulants, n (%)	1 (4.6)	4 (7.6)	1.000
History of PEA, n (%)	1 (4.5)	1 (1.9)	0.503
Supplemental oxygen, n (%)	12 (54.5)	22 (41.5)	0.302
Medication			
Riociguat, n (%)	6 (27.3)	10 (18.9)	0.419
Diuretics, n (%)	11 (50.0)	21 (39.6)	0.408
Anticoagulant, n (%)	21 (95.5)	49 (92.5)	1.000
Antipsychotics, n (%)	21 (95.5)	9 (17.0)	< 0.001
Number of antipsychotics	5 (3–6)	0 (0–0)	< 0.001

Values given as mean ± standard deviation, n (percentage), or median (25th − 75th percentile)

PD, psychiatric disorder; PEA, pulmonary endarterectomy; VTE, venous thromboembolism

**Table 2:** Baseline functional, laboratory, hemodynamic, and respiratory parameters

	PD (n = 22)	Non-PD (n = 53)	p value
Symptom and exercise capacity			
Duration from symptom to diagnosis < 1 year, n (%)	11 (50.0)	30 (56.6)	0.601
WHO functional class (I/II/III/IV)	1/4/16/1	0/16/35/2	0.321
6-min walk distance, m (n = 65)	336 (306–429)	387 (320–455)	0.283
Laboratory parameters			
Hemoglobin, g/dl	13.8 ± 1.5	13.5 ± 1.7	0.435
BNP, pg/ml	32 (13–105)	117 (54–373)	0.001
Hemodynamic data			
Heart rate, bpm	74 ± 13	78 ± 15	0.415
Mean RAP, mmHg	7 ± 2	7 ± 3	0.725
Mean PAP, mmHg	40 ± 7	41 ± 9	0.477
Mean PCWP, mmHg	9 ± 3	9 ± 3	0.373
Mean blood pressure, mmHg	82 ± 13	89 ± 16	0.086
SaO_2_, %	87.9 ± 4.9	90.9 ± 4.1	0.009
SvO_2_, %	64.4 ± 5.3	62.7 ± 9.4	0.745
CO, l/min	5.1 ± 1.6	4.3 ± 1.1	0.116
CI, l/min/1.73m^2^	2.9 ± 1.0	2.7 ± 0.7	0.565
PVR, dyne∙s∙cm^− 5^	491 (376–654)	667 (435–792)	0.066
Respiratory function			
Percent vital capacity, % (n = 70)	89.9 ± 16.2	92.5 ± 13.6	0.511
FEV_1.0_%, % (n = 70)	73.7 ± 9.6	72.8 ± 10.3	0.781
%DLCO, % (n = 70)	75.1 ± 13.1	82.5 ± 16.4	0.074
A-aDO_2_, mmHg (n = 72)	46 ± 9	42 ± 10	0.103

Values given as mean ± standard deviation, n (percentage), or median (25th − 75th percentile). WHO functional class expressed as number of patients in individual class

A-aDO_2,_ alveolar-arterial oxygen gradient; BNP, brain natriuretic peptide; bpm, beats per min; CI, cardiac index; CO, cardiac output; %DLCO, diffusing capacity for carbon monoxide; FEV_1.0_%, forced expiratory volume % in 1 s; PAP, pulmonary artery pressure; PCWP, pulmonary capillary wedge pressure; PD, psychiatric disorder; PVR, pulmonary vascular resistance; RAP, right atrial pressure; SaO_2_, arterial oxygen saturation; SvO_2_, mixed venous oxygen saturation, WHO, World Health Organization

### Functional and hemodynamic effects of BPA in PD patients

Patients with and without PD underwent comparable numbers of BPA sessions [5 (4–9) vs. 6 (4–7) sessions, respectively, p = 0.409], with no significant difference in median follow-up duration from the last BPA session to RHC [177 (76–213) vs. 101 (59–203) days, respectively, p = 0.277]. Both groups experienced significant improvements in BNP, World Health Organization functional class, 6-min walk distance, SaO_2_, and A-aDO_2_ after BPA, whereas BNP remained higher in the non-PD group [12 (9–21) vs. 26 (15–55) pg/ml, p < 0.05] (Table 3). Follow-up RHC also demonstrated significant reductions in mPAP and PVR regardless of concomitant PD (Table 3), with similar numbers of patients achieving mPAP < 30 mmHg in the two groups [19 (86.4%) in PD vs. 46 (86.8%) in non-PD, p = 0.960].

**Table 3:** Functional, laboratory, and hemodynamic parameters before and after BPA

	PD (n = 22)			Non-PD (n = 53)		
	Pre-BPA	Post-BPA	p value	Pre-BPA	Post-BPA	p value
WHO functional class (I/II/III/IV)	1/4/16/1	4/17/1/0	< 0.001	0/16/35/2	12/38/3/0	< 0.001
6-min walk distance, m (n = 56)	336 (300–434)	480 (389–515)	0.004	389 (334–464)	461 (396–536)	< 0.001
BNP, pg/ml (n = 74)	32 (13–105)	12 (9–21)	0.011	119 (57–375)^*^	26 (15–55)^**^	< 0.001
Mean PAP, mmHg	40 ± 7	25 ± 8	< 0.001	41 ± 9	24 ± 6	< 0.001
CI, l/min/1.73m^2^	2.9 ± 1.0	2.9 ± 0.6	0.984	2.7 ± 0.7	2.8 ± 0.6	0.288
PVR, dyne∙s∙cm^− 5^	491 (376–654)	215 (175–261)	< 0.001	667 (435–792)	229 (170–350)	< 0.001
SaO_2_, %	87.9 ± 4.9	92.8 ± 5.2	0.002	90.9 ± 4.1^*^	94.3 ± 3.2	< 0.001
SvO_2_, %	64.4 ± 5.3	69.3 ± 8.9	0.005	62.7 ± 9.4	70.8 ± 5.8	< 0.001
A-aDO_2_, mmHg (n = 68)	45 ± 9	27 ± 13	< 0.001	42 ± 10	26 ± 11	< 0.001

Values given as mean ± standard deviation, n (percentage), or median (25th − 75th percentile). WHO functional class expressed as number of patients in individual class. ^*^p < 0.05 vs. pre-BPA in PD group. ^**^p < 0.05 vs. post-BPA in PD group

A-aDO_2_, alveolar-arterial oxygen gradient; BNP, brain natriuretic peptide; BPA, balloon pulmonary angioplasty; CI, cardiac index; PAP, pulmonary artery pressure; PD, psychiatric disorder; PVR, pulmonary vascular resistance; SaO_2_, arterial oxygen saturation; SvO_2_, mixed venous oxygen saturation; WHO, World Health Organization

### Impact of BPA on QOL score in PD patients

**Fig. 2:**
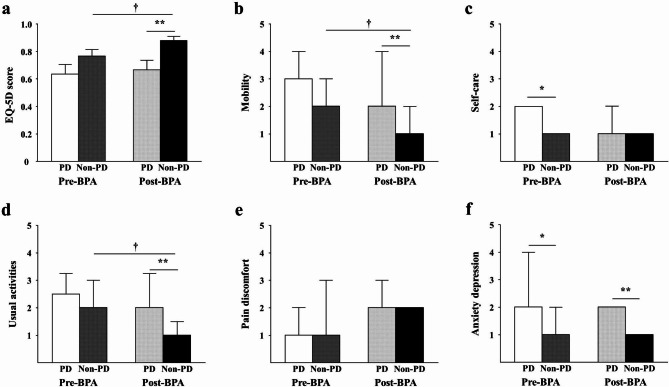
EQ-5D scores and components in PD and non-PD groups before and after BPA. Bar graphs show EQ-5D score (**a**) and its components, including mobility (**b**), self-care (**c**), usual activities (**d**), pain discomfort (**e**), and anxiety depression (**f**) according to PD status before initial BPA and after last BPA procedure. Bars of EQ-5D score demonstrate mean values with standard error. Bars of each component of EQ-5D score represent median values with interquartile range. Left-side bars correspond to pre-BPA QOL status and right-side bars correspond to post-BPA QOL status. * p < 0.05 vs. PD group before BPA. ** p < 0.05 vs. PD group after BPA. **†** p < 0.05 vs. non-PD group before BPA. BPA, balloon pulmonary angioplasty; EQ-5D, European Quality of Life Five Dimension; PD, psychiatric disorder; QOL, quality of life

The EQ-5D score was calculated in 27 patients (10 PD, 17 non-PD) before and after BPA. Before BPA, patients with PD tended to have lower EQ-5D score compared with patients without PD (0.63 ± 0.22 vs. 0.77 ± 0.19, p = 0.102) (Fig. 2a), which was explained by significantly poorer self-care and greater anxiety (Fig. 2b-f). The EQ-5D score after BPA sessions improved significantly in the non-PD group (from 0.77 ± 0.19 to 0.88 ± 0.13, p < 0.001) but remained unchanged in the PD group (from 0.63 ± 0.22 to 0.67 ± 0.22, p = 0.770), with a significantly higher post-BPA EQ-5D score in the non-PD group (0.88 ± 0.13 vs. 0.67 ± 0.22, p = 0.012) (Fig. 2a). This discrepancy was mainly attributable to lack of improvements in mobility, usual activities and anxiety depression following BPA in the PD group compared with the non-PD group (Fig. 2b-f).

## Discussion

This study showed that 29.3% of patients with inoperable CTEPH who underwent BPA had concomitant PD. Before BPA, although PD group had a similar PH severity and exercise capacity, they tended to have unfavorable QOL status. BPA improved pulmonary hemodynamics, laboratory parameters, exercise tolerance, and A-aDO_2_ irrespective of co-existing PD, but did not improve QOL profiles in patients with PD.

The present study demonstrated that 29.3% of patients with inoperable CTEPH treated with BPA had concomitant PD. A previous single-center cohort study by Tajima et al. reported that approximately 10% of patients with CTEPH were complicated by PD [10], and Dering et al. also demonstrated that almost one-third of patients with CTEPH had concurrent psychological disorders [20, 21]. Furthermore, Suzuki et al. found that the prevalence of schizophrenia in patients with CTEPH (7.3%) was 11-fold higher than in the general population [9]. The higher prevalence of psychosis in patients with CTEPH relative to the general population indicates a certain association between CTEPH and PD [22].

There are several possible mechanisms for the relationship between CTEPH and PD. First, enhanced venous stasis due to immobilization related to the disease conditions (e.g., depression) or sedation, and obesity mediated by poor physical activity and drug-induced metabolic abnormalities, could lead to the development of VTE in patients with PD [8, 23]. A population-based cohort study accordingly found that schizophrenia carried a two-fold independent risk for VTE [24]. Second, some antipsychotics, including phenothiazines, clozapine, and risperidone, were shown to be related to serotonin-induced platelet aggregation and elevated antiphospholipid antibodies, contributing to increased thrombogenesis [8, 25]. Indeed, a large primary care cohort in the United Kingdom demonstrated that a prescription of antipsychotic drugs was associated with a 1.3-fold increased risk of VTE [23]. Furthermore, atypical symptoms in patients with PD might result in an underestimation and delayed diagnosis of acute VTE, leading to the subsequent development of CTEPH [26]. In fact, despite an enhanced risk of VTE in patients with PD, the prevalence of acute VTE was comparable between PD and non-PD groups in the present study.

The current results showed that patients with CTEPH and PD tended to be younger than those without PD, in accordance with a recent study in 107 patients with CTEPH [20]. Pre-procedural mPAP was similar between PD and non-PD groups, as shown in the previous studies [10, 27]. In contrast, although statistically insignificant, PD patients tended to have lower PVR compared with their non-PD counterparts, which was different from the previous studies [10, 27]. This discrepancy might be explained by the relatively small study population in the present work. After a series of BPA sessions, mPAP was significantly improved to a comparable level in both groups, suggesting that BPA could have favorable effects on pulmonary hemodynamics, irrespective of concomitant PD.

Recent strategic and procedural advancements of BPA contribute to an improvement in QOL [13, 28–30] beyond prognostic benefit [12, 31, 32]. Daroncha et al. showed a prominent improvement in the 36-item Short Form questionnaire after BPA [28], and Hoole et al. also demonstrated that the Cambridge Pulmonary Hypertension Outcome Review QOL questionnaire was ameliorated in 30 patients with CTEPH treated with BPA [29]. Moreover, Miura et al. found a favorable effect of extensive BPA after achieving mPAP < 30 mmHg on QOL score, beyond hemodynamic improvement [30]. In contrast to these studies however, the current results revealed that BPA was less effective in terms of QOL status in patients with PD, regardless of hemodynamic and functional restoration. Depression and anxiety were also associated with poor QOL in a previous study in patients with PH, including CTEPH [11]. The anxiety-depression score was more impaired in the PD group before and after BPA, which could partially explain the differences in QOL scores between the two groups. Furthermore, patients with PD had poorer scores in other QOL domains, including mobility and usual activities after BPA. Given that QOL is determined by a broad spectrum of physical and psychological properties, patients with CTEPH and PD might require additional physical and psychological interventions, in addition to BPA, to improve their QOL.

The present study revealed a high prevalence of PD in patients with CTEPH, and showed similar PH severity compared with those without PD. Although the frequency of mental disorders in patients with CTEPH varied among individual studies [9, 10, 20], a higher prevalence of PD among patients with CTEPH rather than the general population suggest that patients with PD may be a high-risk group for developing CTEPH, which should be clarified in larger study population. In addition, the present study demonstrated that patients with CTEPH and preexisting PD did not benefit from BPA in terms of QOL, irrespective of favorable effects on hemodynamic and functional parameters, possibly because the beneficial effects of BPA on QOL were canceled out by mental and physical factors. Further studies are warranted to determine if BPA combined with physical and psychiatric interventions could improve QOL status in patients with CTEPH and concomitant PD.

This study had several limitations. First, its small-scale, single-center, retrospective observational study design may limit the applicability of the results to other populations. Second, QOL was evaluated in only 27 of the 75 patients, which may limit concluding a difference in the impact of BPA on QOL status according to the presence or absence of PD in CTEPH patients. Third, a depression scale was not conducted in this study to assess mental status, so a detailed analysis of depression is not available. Finally, the prevalence of acute VTE was relatively high compared to the previous report in Japan [33], which might be partially explained by the possibility that we might include “acute” and “acute on chronic” pulmonary embolism and could not precisely distinguish these two phenotypes due to the retrospective nature of the present study. Further studies are therefore required to investigate the long-term effects of BPA on QOL status in a large CTEPH population with PD.

## Conclusions

This study found that 29.3% of patients with inoperable CTEPH who underwent BPA had co-existing PD. PH severity was comparable between PD and non-PD patients, and BPA improved hemodynamic and functional parameters in both groups. In terms of QOL, although the limited number of study population, PD patients were likely to have unfavorable QOL status compared with non-PD patients, and the effect of BPA on QOL was limited in the PD group. These results should be confirmed in further studies with a larger study population.

## Data Availability

The data that support the findings of this study are available from the corresponding author upon reasonable request.

## List of abbreviations

A-aDO_2_: Alveolar-arterial oxygen gradient
BNP: Brain natriuretic peptide
BPA: Balloon pulmonary angioplasty
CTEPH: Chronic thromboembolic pulmonary hypertension
EQ-5D: European Quality of Life Five Dimension
mPAP: Mean pulmonary artery pressure
PD: Psychiatric disorder
PEA: Pulmonary endarterectomy
PH: Pulmonary hypertension
PVR: Pulmonary vascular resistance
QOL: Quality of life
RHC: Right-sided heart catheterization
VTE: Venous thromboembolism
